# Factors influencing HAART adherence among private health care sector patients in a suburb of the Ethekwini Metro

**DOI:** 10.4102/phcfm.v1i1.12

**Published:** 2009-05-06

**Authors:** Panjasaram Naidoo

**Affiliations:** School of Pharmacy and Pharmacology, University of KwaZulu-Natal, South Africa

**Keywords:** adherence, factors, private sector, difficulty in swallowing, Ethekwini Metro

## Abstract

**Background:**

The advent of highly active antiretroviral therapy (HAART) ushered in a new era in the management of the AIDS pandemic with new drugs, new strategies, new vigour from treating clinicians and enthusiasm on the part of their patients. What soon became evident, however, was the vital importance of patient adherence to prescribed medication in order to obtain full therapeutic benefits. Several factors can influence adherence to HIV drug regimens. Many treatment regimes are complex, requiring patients to take a number of drugs at set times during the day, some on a full stomach and others on an empty one. Other factors that could contribute to non-adherence include: forgetting to take medications, cost factor, side effects, incorrect use of drug, social reasons, denial or poor knowledge of drug regime. If the correct regimen is not prescribed and if patients do not adhere to therapy, then the possibility of resistant strains is high. Improving adherence is therefore arguably the single most important means of optimising overall therapeutic outcomes. Although several studies regarding patient adherence have been performed in the public health care sector, data on adherence in patients from the private health care sector of South Africa remain limited. Many factors influence compliance and identifying these factors may assist in the design of strategies to enhance adherence to such demanding regimens. This study aimed to identify these factors among private sector patients.

**Method:**

Descriptive cross-sectional study was conducted among all consenting patients with HIV who visited the rooms of participating private sector doctors from May to July 2005. A questionnaire was administered to consenting participants. Participants who reported missing any medication on any day were considered non-adherent. The data obtained was analysed using SPSS 11.5. A probability value of 5% or less was regarded as being statistically significant. Categorical data was described using frequency tables and bar charts. Pearson's chi-square tests or Fischer's exact tests were used interchangeably as appropriate to assess associations between categorical variables. The study received ethics approval from the University of KwaZulu-Natal's Nelson R Mandela School of Medicine Ethics Committee.

**Results:**

A total of 55 patients completed the questionnaires and 10 patients refused to participate. There was no statistical difference between adherence to treatment and demographics such as age, gender and marital status. In this study 89.1% of patients were classified as non-adherent and reasons for nonadherence included difficulty in swallowing medicines (67.3%) (p = 0.01); side effects (61.8%) (p = 0.03); forgetting to take medication (58.2%) (p = 0.003); and not wanting to reveal their HIV status (41.8%) (p = 0.03). Common side effects experienced were nausea, dizziness, insomnia, tiredness or weakness. Reasons for taking their medicines included tablets would save their lives (83.6%); understand how to take the medication (81.8%); tablets would help them feel better (80.0%); and were educated about their illness (78.2%). The majority of participants (65.5%) were on two nucleoside reverse transcriptase inhibitors (NRTIs) and one non-nucleoside reverse transcriptase inhibitor (NNRTI). All participants that were on a regimen that comprised protease inhibitors and two NRTIs were found to be non-adherent.

**Conclusion:**

Some barriers to adherence among this cohort of private sector patients are similar to those experienced by public sector patients. It will be important for doctors to identify these problems and implement strategies that could improve adherence, e.g. using short message services (SMSs) reminders for those patients prone to forgetting to take their medicines, breaking the tablets into smaller pieces in order to overcome the difficulty of swallowing, if the medication is not available in a liquid form, looking at alternative medication with lesser or more tolerant side effect profiles and greater counselling on the drugs.

## INTRODUCTION

On a global scale, the number of people living with HIV in 2007 was 33.2 million, a reduction of 16% compared with the estimate published in 2006 (39.5 million). In sub-Saharan Africa an estimated 22.5 million adults and children are living with HIV and AIDS. Sub-Saharan Africa remains the most seriously affected region, with AIDS the leading cause of death.^1^

The advent of highly active antiretroviral therapy (HAART) ushered in a new era in the management of patients with AIDS. What soon became evident, however, was the vital importance of patient adherence to prescribed medication in order to obtain full therapeutic benefits.^2^ Adherence to antiretroviral therapy (ART) is a crucial determinant of treatment success. While the ultimate goal of ART is to reduce HIV-related morbidity and mortality, the initial goal is full and durable viral suppression. For most patients, near-perfect (> 95%) adherence is necessary to achieve full and durable viral suppression. In practice, this degree of adherence requires a patient on a twice-daily regimen not to miss or substantially delay more than three doses of antiretroviral medications per month.^3^ Many factors influence compliance and identifying these factors may assist in the design of strategies to enhance adherence to such demanding regimens.^4^

Many treatment regimes are complex and require patients to take a number of drugs at set times during the day, some on a full stomach and others on an empty one. For people who endure tremendous financial hardship and who are unable to work or eat regular meals, the situation is even more challenging. The authors in a study carried out in Cape Town, South Africa, concluded that socioeconomic status had no impact on adherence, and that patients with very little or no financial means can have successful treatment outcomes when the financial barriers to treatment are removed. However, the number of times the medicines were taken was a strong predictor for poor adherence and virological failure.^5^ In another study carried out in a public sector hospital in Soweto, South Africa, the main reasons cited for missing doses among patients were being away from home (30%), difficulty with the dosing schedules (23%), and running out of tablets (12%). Adherence decreased considerably with fear of being stigmatized by the sexual partner (OR = 0.13 95%, CI 0.02-0.70).^6^

If barriers that impede medication adherence go unrecognised and unresolved, the management of HIV and AIDS patients would be compromised with disastrous results in terms of resistance emerging. Resistance to antiretroviral drugs is a concern in the treatment of HIV and AIDS, because once it occurs, it affects patients’ clinical outcome and also reduce patients’ treatment options. While this is clearly a bad situation for the individual, from a public health perspective it is also alarming to have multiple drug-resistant strains of HIV that can be transmitted to non-infected people.^7^ Improving adherence is therefore arguably the single most important means of optimising overall therapeutic outcomes. A study carried out among primary care public sector patients in South Africa demonstrated that with a standard approach of preparing patients and strategies to enhance adherence, patients on antiretroviral medication can be retained in a resource-limited setting.^8^

Although studies have been carried out to establish the extent of adherence in public sector patients, there is limited adherence data from patients in the private health care sector of South Africa, particularly in KwaZulu-Natal – the province with the highest HIV and AIDS prevalence rates. Furthermore, in anecdotal reports from private sector doctors there was an indication that many of their patients are non-adherent. They maintain that they follow the guidelines prescribed for optimum care of these patients but the desired therapeutic outcome is not evident in the majority of their patients. Therefore, based on the knowledge that ART has been available in the private health care sector since 1996, it was decided that a study be conducted among patients visiting private sector doctors to identify factors that influence adherence to ART.

## METHOD

### Study design

A cross-sectional, descriptive study was conducted.

### Sampling

Doctors working within a 2 km radius of an area to the south of the Ethekwini region of KwaZulu-Natal were asked to participate in the study. These doctors had to be managing HIV and AIDS patients. The doctors were practicing in an area that saw South African patients from both African and Indian origin, whose socioeconomic status ranged from very poor to very wealthy. Some lived in formal housing and others in informal housing. The study was conducted in a private and enclosed area of each of the medical practices selected.

The sampling frame included all consenting patients with HIV and AIDS visiting the selected medical practices during the research period, identified as the months of May to July 2005. In addition, to be eligible, patients had to be on ART. An appointment register was available to the researchers on the day of the study. The HIV and AIDS patients who were receiving treatment and who were visiting the practices on that specific day were highlighted in the register by the doctor. The researchers made contact with these patients on their arrival at the doctors’ rooms for their appointments and explained the study to them. Those patients who were willing to participate and who signed a consent form were enrolled in the study.

### Study variables and instrument

Previously piloted self-reported questionnaires containing both open-ended and closed questions were developed by the researchers. These questionnaires, available in English and IsiZulu, were administered by the researchers to the participants in their preferred language. Medical charts of the participants were viewed to confirm the current HIV treatment regimen. Participants who reported missing any medication on any day in a month were considered non-adherent (95% adherence level). The instrument was divided into two sections, namely demographics and medicine taking (reasons for not taking medication, side effects and perceived positive outcomes with respect to whether the medicines were working or not working for them).

### Statistical analysis

The data obtained was analysed using SPSS Version 11.5. A probability value of 5% or less was regarded as being statistically significant. Categorical data was described using frequency tables and bar charts. Pearson's chi-square tests or Fischer's exact tests were used interchangeably as appropriate to assess associations between categorical variables. The study received ethics approval from the University of KwaZulu-Natal's Nelson R Mandela School of Medicine Ethics Committee.

## RESULTS

Four doctors participated in the study and a total of 55 patients were enrolled. Of the 55 patients that participated in the study, 89.1% were classified as non-adherent.

The majority of participants n = 37 (67%) were female and 83.6% of participants were between the ages of 20 to 39 years, of which 86.9% were non-adherent.

The most common HAART regimens were: ***regimen one***: zidovudine, lamivudine and efavirenz (45.5%); ***regimen two***: lamivudine, stavudine and efavirenz (20.0%); and ***regimen three***: lopinavir, ritonovir, zidovudine and lamivudine (12.7%). The majority of participants (65.5%) were on two nucleoside reverse transcriptase inhibitors (NRTIs) and one non-nucleoside reverse transcriptase inhibitor (NNRTI). All participants on regimen three (comprising protease inhibitors (PIs) and two NRTIs), were found to be non-adherent.

### Reasons for not taking medication

Respondents offered many reasons for not taking their medication, with significant findings relating to the difficulty experienced in swallowing the medication, side effects of the medication, forgetting to take their medication and reasons relating to stigma. Interestingly, 9.1% of respondents did not take their medicines because they were not part of the decision making about their illness with the doctor. (Table 1).

**TABLE 1:** Reasons for not taking medication (n = 55)

REASONS FOR NOT TAKING MEDICATION	PATIENTS (%)	P VALUE
Difficulty in swallowing medicine	67.3	0.01
Side effects	61.8	0.03
Forgot to take medicine	58.2	0.003
Not reveal HIV status	41.8	0.03
Too many tablets to take	32.7	Not significant
Difficult to take specific times of day	23.6	Not significant
Falling asleep	20	Not significant
Feeling sad	20	Not significant
Too busy	18.2	Not significant
Did not receive medicines from pharmacy	12.7	Not significant
Too many directions to take	10.9	Not significant
Difficulty in acquiring the medicine	10.9	Not significant
Not part of decision making	9.1	Not significant
Ran out of medicine	9.1	Not significant
**Open-ended responses**		
Husbands not allowing wives to take medication	4.2	Not significant
Felt better therefore stopped taking medicines	2.1	Not significant
Had body aches in the morning therefore not taking morning dose	2.1	Not significant

### Side effects

When respondents were asked about whether they experienced any side effects, over 55% of respondents reported nausea, dizziness and insomnia as side effects with a small percentage (1.8%) reporting ketoacidosis and kidney stones as a side effect (Table 2).

**TABLE 2:** Side effects experienced (n = 55)

SIDE EFFECT	PATIENT (%)
Nausea	67.3
Dizziness	56.4
Insomnia	56.4
Fatigue and weakness	49.1
Numbness	47.3
Diarrhoea	45.5
Anaemia	16.4
Abdominal pain	16.4
Hepatitis	3.6
Diabetes	3.6
Lipodystrophy	3.6
Ketoacidosis	1.8
Kidney stones	1.8

### Reasons for taking medication

**FIGURE 1:**
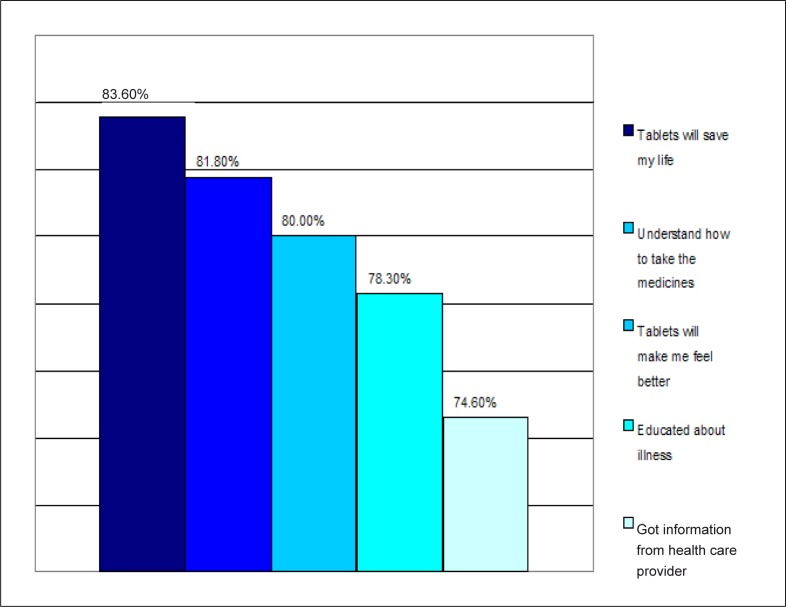
Reasons for taking medication

Figure 1 describes the reasons why participants took their medication, with 83.6% stating that they take their medicines because the tablets will save their lives and 74.6% being influenced by the information provided by their health care provider.

## DISCUSSION

The majority of the participants surveyed in the four participating medical practices were non-adherent. Some of the reasons cited were significant, such as side effects experienced, difficulty in swallowing, forgetting to take their medication and stigma. The majority of the participants who were between the ages of 20 to 39 were found to be non-adherent. This finding, though not statistically significant, is supported by a presentation and study carried out by Valerie Stone, in which it was indicated that the younger age (< 35 years) is a patient factor predictive of nonadherence, and that young females were more non-adherent.^9^ Another study also found that moderate and poor adherence were independently associated with younger age.^10^

The most problematic dosage regimen was the one that had protease inhibitors (PIs), that is, lopinavir and ritonavir, together with zidovudine and lamivudine. The total number of tablets taken in this regimen was greater than seven per day and may have contributed to participants being non-adherent. Several studies have shown that regimen complexity significantly affects non-adherence.^11, 12, 13^

As a result of their many adverse effects, drug interactions and food restrictions,^10, 14, 15, 16^. HAART regimens, especially those containing PIs, have been implicated in promoting non-adherence.

It is widely known that side effects to ART contribute to non-adherence and discontinuation of HAART.^11, 17, 18^ This study demonstrated that 61.8% of participants did not take their medication due to side effects, with nausea being the most common side effect experienced by participants. This is consistent with other studies that show that nausea increases the risk for non-adherence regardless of the time the participants were on medication.^19^ Insomnia and dizziness were also commonly experienced by participants who were on efavirenz. This is a well-documented side effect for people with HIV who are on long-term efavirenz therapy^20^ and on a HAART regimen.^19^

This study also found that participants frequently forgot to take their medication, which is not unique to this study but has been reported previously^4, 9, 11, 17^ as one of the most common or frequent reasons for non-adherence. Reminders such as short message services (SMSs) on cell phones, alarm wrist watches or timer settings should be encouraged to assist patients in remembering to take their medicines on time.

An interesting finding in this study was the association of difficulty in swallowing and being non-adherent. Participants in this study experienced difficulty in swallowing their medication and were therefore not able to take them. This reason for non-adherence has not been previously cited and perhaps should be looked at seriously when dosage forms are being prescribed or dispensed to patients. Pill size was reported as one of the attributes that to some degree impacts on adherence.^21^

This study also reported consistent findings with other studies^22^ in terms of stigmatisation and adherence to therapy in that participants did not take their medication because they did not want to reveal their HIV status. Therefore, it is important when counselling is provided to people with HIV that adherence and disclosure not be separated. Interventions targeted at adherence should seek to help the patient to concurrently manage disclosure of their seropositivity and its consequences.^23^ Some of the reasons provided by respondents as to why they take their medication confirm that when they are educated about their illness, have information from their health care provider about their illness or medicines, or if they understand how to take their medicines patients feel confident to take the medication. However, when they were not part of the decision making, some of these patients did not take their medication as reported. Barriers to adherence such as patient factors, health care providers themselves and the health care system need special attention in order to improve adherence. Strategies such as educating patients, improving communication between patients and health care providers, improving dose scheduling, providing drugs with less adverse effects and improving accessibility to health care are of paramount importance in the battle against non-adherence.^24^ While patients are ultimately responsible for taking their medication, good communication, involving the patient in decision making about their care and simplifying drug regimens go a long way in improving it.^24^

### Limitations of the study

The sample size in this study was small and therefore it does not allow a general statement to be made about all private sector patients in South Africa.

### Conclusion and recommendations

The results from this study indicate that adherence to ART is a problem among private health care sector patients in KwaZulu-Natal, and that barriers to adherence do exist. These barriers to adherence are similar to those cited by public sector patients. The researchers recommend that a larger study, with a bigger sample size, be carried out a much wider area. In order to validate the authenticity of the participants’ responses, the medical charts should be used to confirm whether they are adherent or not. The clinical outcomes of the patients may be used as an indicator. Reported interventions that improve adherence such as pill boxes, SMS reminders, alarms and pill counts should be implemented. Dosage forms should be prescribed according to individual preferences; however, when not available, breaking the tablets carefully into smaller sizes if they are scored should be encouraged and counselled on, so that the entire dose is taken.

## References

[1] UNAIDS AIDS Epidemic update [homepage on the Internet]. 2007 [cited 2008 Mar 16]. Available from: http://data.unaids.org/pub/EPISlides/2007/2007_epiupdate_en.pdf.

[2] US Dept of Veterans Affairs Adherence to HIV antiretroviral therapy. Implications of adherence. [homepage on the internet]. No date [cited 2009 Feb 28]. Available from: http://www.hiv.va.gov/vahiv?page=pr-kb-00&kb=kb-03-02-09&tp=Antiretroviral%20Therapy&tpage=prtop04-00-rr&sec=03.

[3] Maggiolo F, Ripamonti D, Arici C, et al Simpler regimens may enhance adherence to antiretrovirals in HIV-infected patients. HIV Clin Trials. 2002;3(5):371–378. 12407486 10.1310/98b3-pwg8-pmyw-w5bp

[4] Orrell C, Bangsberg DR, Badri M, Wood R. Adherence is not a barrier to successful antiretroviral therapy in South Africa. AIDS. 2003;17(9):1369–1376. 12799558 10.1097/00002030-200306130-00011

[5] Nachega JB, Stein DM, Lehman DA, et al Adherence to antiretroviral therapy in HIV-infected adults in Soweto, South Africa. AIDS Res Hum Retroviruses. 2004;20(10):1053–1056 15585095 10.1089/aid.2004.20.1053

[6] Tsasis P. Adherence assessment to highly active antiretroviral therapy. J Assoc Nurses AIDS Care. 2001;15(3):109–115. 10.1089/108729101750123544 11313023

[7] Coetzee D, Boulle A, Hildebrand K, Asselman V, Van Cutsem G, Goemaere E. Promoting adherence to antiretroviral therapy: The experience from a primary care setting in Khayelitsha, South Africa. AIDS. 2004;18(3):S27–S31. 15322481 10.1097/00002030-200406003-00006

[8] Howard AA, Arnstein JH, Stone VE, et al A prospective study of adherence and viral load in a larger multi-centre cohort of HIV-infected women. AIDS. 2002;16(16):2175–2182. 12409739 10.1097/00002030-200211080-00010

[9] Carrieri MP, Leport C, Protopopescu C, et al Factors associated with non-adherence to highly active antire troviral therapy: A 5-year follow-up analysis with correction for the bias induced by missing data in the treatment maintenance phase. JAIDS. 2006;41(4):477–485. 16652057 10.1097/01.qai.0000186364.27587.0e

[10] Jones DL, Ishii M, Perriere LA, et al Influencing medication adherence among women with AIDS. Journal of International Association of Physicians in AIDS Care. 2003; 15:463–474. 10.1080/0954012031000134700 14509861

[11] Horne R, Buick D, Fischer M, Leake H, Cooper V, Weinman J. Doubts about necessity and concerns about adverse effects: Identifying the types and beliefs that are associated with non-adherence to HAART. Int J STD and AIDS. 2004;15(1):38–44. 14769170 10.1258/095646204322637245

[12] Chesney M. Adherence to HAART regimens. J Assoc Nurses AIDS Care. 2003;17:169–175. 10.1089/108729103321619773 12737640

[13] Van Heeswijk RP, Veldman A, Mulder JW, et al Combination of protease inhibitors for the treatment of HIV-1 infected patients: A review of pharmacokinetics and clinical experience. Antiv Ther. 2001;6(4):201–229. 11878403

[14] Trotta MP, Ammassari A, Melzi S, et al Treatment related factors and highly active anti-retroviral therapy adherence. JAIDS. 2002;31(3):S128–S131. 12562035 10.1097/00126334-200212153-00008

[15] Proctor VE, Tesfa A, Tompkins DS. Barriers to adherence to highly active antiretroviral therapy as expressed by people living with HIV/AIDS. J Assoc Nurses AIDS Care. 1999; 13(9): 535–544 10.1089/apc.1999.13.535 10813033

[16] Nachega JB, Lehman DA, Hlatshwayo D, Mothopeng R, Chaisson RE, Karstaedt AS. HIV/AIDS and anti-retroviral treatment knowledge, attitudes, beliefs, and practices in HIV infected adults in Soweto, South Africa. JAIDS. 2005;38(2):196–201. 15671805 10.1097/00126334-200502010-00011

[17] Heath KV, Singer J, O'Shaughnessy MV, Montaner JS, Hogg RS. Intentional non-adherence due to adverse symptoms associated with antiretroviral therapy. J Acquir Immune Defic Syndr. 2002;31(2):211–217. 12394800 10.1097/00126334-200210010-00012

[18] Ammassari A, Murri R, Pezzotti P, et al Self-reported symptoms and medication side effects influence adherence to highly active antiretroviral therapy in persons with HIV infection. JAIDS. 2001;28(5):445–449. 11744832 10.1097/00042560-200112150-00006

[19] Fumaz CR, Munoz-Moreno JA, Molto J, et al Long-term neuro-psychiatric disorders on efavirenz-based approaches: Quality of life, psychologic issues, and adherence. JAIDS 2005;38(5):560–565. 15793366 10.1097/01.qai.0000147523.41993.47

[20] NAM, AIDSMAP Resources Michael Carter: Once daily treatment not a golden bullet for improved adherence, multiple factors important, [hompage on the Internet]. No date [cited 2004 June 23]. Available from: http://www.aidsmap.com/en/news/C6F86FA7-A4D3-40FA-BB89-B788594556BA.asp.

[21] Reynolds NR, Testa MA, Mark LG, et al Factors influencing medical adherence beliefs and self-efficacy in persons naïve to ARV therapy. AIDS. 2004;8:141–150. 10.1023/B:AIBE.0000030245.52406.bb 15187476

[22] Peretti-Watel P, Spire B, Pierret J, Lert F, Obadia Y. VESPA Group Management of HIV-related stigma and adherence to HAART: Evidence from a large representative sample of outpatients attending French hospitals (ANRS-EN12-VESPA 2003). Journal of International Association of Physicians in AIDS Care. 2006;18(3):254–261. 10.1080/09540120500456193 16546787

[23] Chia YC. Understanding patient management: The need for medication adherence and persistence. Malaysian Family Physician 2008;3(1):2–6. 25606104 PMC4267031

